# Viremia Trajectories of HIV in HIV-Positive Women in the United States, 1994-2017

**DOI:** 10.1001/jamanetworkopen.2019.3822

**Published:** 2019-05-17

**Authors:** Seble G. Kassaye, Cuiwei Wang, Joanne Michelle F. Ocampo, Tracey E. Wilson, Kathryn Anastos, Mardge Cohen, Ruth M. Greenblatt, Margaret A. Fischl, Igho Otofukun, Adaora Adimora, Mirjam-Colette Kempf, Gerald B. Sharp, Mary Young, Michael Plankey

**Affiliations:** 1 Department of Medicine, Georgetown University Medical Center, Washington, DC; 2 Department of Community Health Sciences, Downstate Medical Center School of Public Health, State University of New York, Brooklyn; 3 Departments of Medicine and Epidemiology & Population Health, Albert Einstein College of Medicine, Montefiore Health Systems, New York, New York; 4 Ruth M. Rothstein CORE Center, Stroger Hospital, Cook County Bureau of Health Services, Chicago, Illinois; 5 Department of Clinical Pharmacy, Schools of Pharmacy and Medicine, University of California, San Francisco; 6 Department of Medicine, University of Miami Miller School of Medicine, Miami, Florida; 7 Department of Medicine, Emory University, Atlanta, Georgia; 8 Department of Medicine, University of North Carolina at Chapel Hill; 9 School of Nursing, University of Alabama at Birmingham, Birmingham; 10 National Institute of Allergy and Infectious Diseases, National Institutes of Health, Bethesda, Maryland

## Abstract

**Importance:**

Viral suppression of HIV is an important treatment goal to decrease morbidity, mortality, and risk of transmission to others.

**Objective:**

To characterize longitudinal HIV viral load outcomes among women enrolled in the Women’s Interagency HIV Study (WIHS).

**Design, Setting, and Participants:**

A prospective cohort study of HIV-positive women with semiannual study visits and a minimum of 5 follow-up visits was conducted from 1994 to 2017. The WIHS sites included in this analysis are in Brooklyn and Bronx, New York; Chicago, Illinois; San Francisco, California; and Washington, DC.

**Main Outcomes and Measures:**

Women were categorized into groups based on their probability of achieving viral load suppression below 200 copies/mL using logistic trajectory modeling. Multinomial regression analysis was used to identify factors associated with placement in the group with the highest probability of viremia.

**Results:**

At baseline, the mean (SD) age of the 1989 women was 36.9 (8.0) years, mean CD4^+^ T-lymphocyte count was 467/mm^3^, median (interquartile range) HIV RNA was 6200.0 (384.5-41 678.0) copies/mL, and 1305 women (65.6%) were African American. Three trajectory groups were identified with low (568 [28.6%]), intermediate (784 [39.4%]), and high (637 [32.0%]) probability of viremia above 200 copies/mL. The mean (SD) cumulative years of viral suppression were 18.7 (4.0) years, 12.2 (3.1) years, and 5.8 (2.9) years in the respective groups. Factors associated with high probability of viremia included younger age (odds ratio [OR]. 0.99; 95% CI, 0.98-0.99; *P* = .03), African American race (odds ratio [OR], 2.43; 95% CI, 1.75-3.37), *P* < .001), Hispanic race/ethnicity (OR, 1.50; 95% CI, 1.03-2.19; *P* = .04), increased levels of depressive symptoms (OR, 1.17; 95% CI, 1.01-1.36; *P* = .03), drug use (OR, 1.23; 95% CI, 1.01-1.51; *P* = .04), lower CD4+ T-lymphocyte counts (OR, 95% CI, 0.82; 0.80-0.85; *P* < .001), and unstable housing (OR, 1.25, 95% CI, 1.03-1.50; *P* = .02). Between 2015 and 2017, 71.2% of women demonstrated sustained viral suppression: 89.6% (310 of 346) of those with low viremia, 83.4% (346 of 415) with intermediate, and 35.2% (112 of 318) with high probability of viremia.

**Conclusions and Relevance:**

This longitudinal approach suggested that the probability of viremia decreased substantially over time for most participants, including among women with earlier histories of incomplete viral suppression. The findings from this study suggest that continued efforts are needed to address mental health, social, behavioral and structural factors that were identified as associated with high probability of HIV viremia over time.

## Key Points

**Question:**

How do longitudinal viral trajectories vary among women with HIV?

**Findings:**

In a cohort study of 1989 women, 3 trajectories were identified with low (28.6%), intermediate (39.4%), and high (32.0%) probability of viremia. Although younger age, African American or Hispanic race/ethnicity, depression symptoms, drug use, and unstable housing were associated with a high probability of viremia, between 2015 and 2017, 71.2% of women achieved sustained viral suppression, including 35.2% of those in the group with a high probability of viremia.

**Meaning:**

Despite substantial demonstrated success in decreasing HIV viremia substantially over time for most of the women, continued efforts appear to be needed to address mental health, social, behavioral, and structural factors that continue to be associated with the high probability of sustained viremia.

## Teaser

This cohort study examines the changes in viral suppression among HIV-positive women in the United States from 1994 to 2017.

## Introduction

The HIV care continuum is a conceptual model that depicts the distribution of HIV-positive individuals from diagnosis to treatment and viral suppression and serves as a public health tool to estimate the effectiveness of HIV services.^1^ Universal and early treatment are now recommended for all HIV-positive individuals to lower HIV transmission and improve survival.^2,3,4,5,6^ Clinicians now recommend early treatment,^7^ and these advances have led to an increase in life expectancy among HIV-positive individuals in the United States.^8^ Using a cross-sectional approach, 2014 US national surveillance data found that approximately 57% of HIV-positive individuals demonstrated viral suppression.^1,9^ Crepaz et al^10^ analyzed data from the same 1-year period and found that only 47% of the patients maintained viral suppression through multiple HIV RNA tests during 2014, highlighting the limitations of the cross-sectional approach when depicting the HIV care continuum. Other analyses have demonstrated heterogeneity in viral suppression, with lower rates of viral suppression in nonmetropolitan urban and rural areas.^11^

The evolving treatment guidelines, variations in viral suppression, and increased policy focus on achieving viral suppression prompted our previous study that examined the longitudinal viral suppression in the Metropolitan Washington, DC, Women’s Interagency HIV Study (WIHS).^12^ Using a group-based trajectory analysis we identified 3 distinct longitudinal viremia trajectory patterns: high, intermediate, and low probability of viral suppression.^12^ This initial study was limited to the mid-Atlantic WIHS participants. We expanded this analysis to the national WIHS cohort that has representation from across the United States to determine whether similar longitudinal patterns of viremia emerge. The current goals of treatment aim to decrease transmission and improve survival and require long-term adherence and viral suppression. The objective of this study was to identify trends in viral suppression and factors associated with the viral trajectory groups that are modifiable to improve viral suppression.

## Methods

### Study Population

The WIHS is an ongoing multicenter, prospective, observational interval, cohort study. Original research sites were in Bronx and Brooklyn, New York; Washington, DC; Los Angeles and San Francisco, California; and Chicago, Illinois, with 3 enrollment waves in 1994-1995, 2000-2001, and 2010-2013.^13,14^ New sites were added and additional enrollment took place from 2013 to 2015 in Atlanta, Georgia; Chapel Hill, North Carolina; Miami, Florida; Birmingham, Alabama; and Jackson, Mississippi.^15^ A total of 3701 HIV-positive women were enrolled from clinics and the community. Only original sites and participants with at least 5 visits were included in this analysis to avoid bias owing to unavailable data from the Los Angeles site that closed in 2013 and shorter duration of follow-up at the new sites. This study followed the Strengthening the Reporting of Observational Studies in Epidemiology (STROBE) reporting guideline for cohort studies. Institutional review boards from all collaborating institutions approved the WIHS protocol, and all women provided written informed consent to participate in the study. Participants receive modest remuneration to compensate for time and travel.

Visit windows are at a fixed 6-month interval, and follow-up visits are scheduled approximately every 6 months (3.5-8.5 months are allowed between visits). Participants provide data about sociodemographic characteristics, sexual behaviors, substance use, health care use, antiretroviral therapy, comorbidities, and disease outcomes.^13,14,15^ Participants undergo targeted physical examinations and provide biological specimens. CD4^+^ T-lymphocyte counts and HIV RNA levels are measured among HIV-positive women. Treatment of HIV is not provided as part of the study and reflects the prescribing practices of local health care professionals. This present analysis was limited to HIV-positive women who attended 5 or more visits during follow-up.

### Outcomes

The primary outcome of interest was the plasma HIV RNA level or viral load measured using standard commercial HIV RNA assays at each semiannual visit and values of 200 copies/mL or lower were classified as suppressed. Group-based trajectory modeling identified patterns of viral suppression over the course of this study. Our analyses are conducted at the group level so we do not censor based on loss to follow-up for the group. Cumulative viral load suppression-years were calculated for each individual at each visit by summing suppression values (1 or 0) across all visits and dividing by 2 to account for semiannual visits. Mortality outcomes reflected all-cause mortality, with death ascertained by obtaining a death certificate on notification of a death and/or periodic systematic searches of the National Death Index to capture unreported deaths.^16,17^

### Covariates

Covariates included race/ethnicity, age, educational level, recreational and drug use, and alcohol intake (dichotomized as 0-7 or >7 drinks per week). Clinical and laboratory factors included CD4^+^ T-lymphocyte counts (enumerated using standard-flow cytometry protocols), symptoms of depression (Center for Epidemiologic Studies Depression Scale [CES-D], with a score ≥16 indicating depression and <16 indicating no depression), HIV therapy (no therapy; antiretroviral therapy [ART], including monotherapy or combination therapy; and combination antiretroviral therapy [cART], including at least 3 antiretrovirals from at least 2 drug classes based on the Department of Health and Human Services 2008 guidelines^18^), and HIV acquisition risk category (intravenous drug use, heterosexual, transfusion, and no identified risk). The analysis also accounted for enrollment wave and site.

### Statistical Analysis

#### Group-Based Trajectories

We used a logistic trajectory model to identify groups with similar longitudinal patterns of viral suppression with HIV RNA detection above or below 200 copies/mL as the binary outcome at each visit. The number of groups was selected based on statistical criteria and prior knowledge of the viremia patterns that exist within the data set (detailed in eTable in the Supplement).^19^ We compared the Bayesian information criteria for a number of groups and polynomial order (0, intercept; 1, linear; 2, quadratic; 3, cubic) to find the model with the best fit based on the lowest Bayesian information criteria. Group–based modeling assigned each woman a posterior probability of belonging to a particular group based on her pattern of HIV RNA levels across visits. The maximum probability rule assigned each individual to a group where the posterior probability of membership was the highest. We used the mean posterior probability of greater than 0.70 to assess goodness of fit.

#### Generalized Linear Modeling

Generalized linear modeling (PROC GENMOD) for repeated measures with generalized estimating equations explored the association between viral trajectory groups and covariates. The model used the multinomial distribution and cumulative logit link function to derive the cumulative probability of going from low to intermediate to high viremia adjusted for covariates. The multivariate analysis included variables with 2-tailed *P* < .05 in the univariate analysis. We conducted an additional analysis to determine whether changing the viral load cutoff to 1000 copies/mL would alter the number of groups assigned and proportions assigned to each group (eFigure in the Supplement). In addition, we plotted the mean cumulative years of viral load suppression for each group (mean years per participant per group). SAS, version 9.2 (SAS Institute Inc) was used for all statistical analyses.

## Results

**Figure 1.**
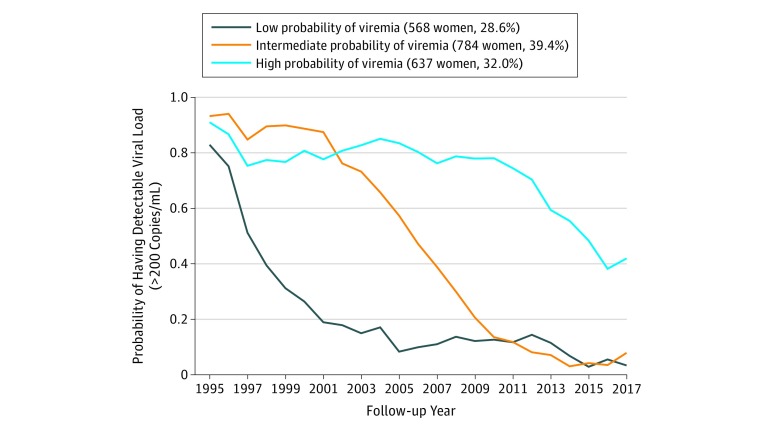
Viral Trajectories for HIV-Positive Women in the Women’s Interagency HIV Study by Probability of HIV RNA Greater Than 200 Copies/mL Group-based trajectory analysis identified 3 distinct viral trajectories among participants in the Women’s Interagency HIV Study. The group with low probability of viremia using a viremia cutoff of greater than 200 copies/mL consisted of 568 women, representing 28.6% of the total population; 784 women (39.4%) were in the intermediate probability of viremia group, and 637 women (32.0%) had high probability of viremia over the 23-year period.

At baseline, among 1989 HIV-positive women the mean (SD) age was 36.9 (8.0) years, mean CD4^+^ T-lymphocyte count was 467/mm^3^, median (interquartile range) HIV RNA was 6200.0 (384.5-41 678.0)copies/mL, and 1305 women (65.6%) were African American (Table 1). These women contributed 26 463 person-years from 1994 to 2017. HIV viral load data were missing for 1512 of 49 168 visits (3.1%). The trajectory analysis identified 3 HIV viral load trajectory groups with a cubic polynomial shape: those with low probability of viremia (low viremia) greater than 200 copies/mL (568 of 1989 [28.6%]), intermediate probability of viremia (intermediate viremia: 784 [39.4%]), and high probability of viremia (high viremia: 637 [32.0%]) (Figure 1) with high mean posterior probabilities for the low (0.92), intermediate (0.75), and high (0.88) viremia groups, demonstrating high precision in the number of groups that best fit the data and assignment of women into the trajectory groups.^18^

**Table 1.** Baseline Characteristics by HIV Viral Trajectory Group Among HIV-Positive Women With More Than 4 Visits

Characteristic	No. (%)			
	Overall	HIV Viral Trajectory Group^a^		
		Low Probability of Viremia	Intermediate Probability of Viremia	High Probability of Viremia
No. (%)	1989	568 (28.6)	784 (39.4)	637 (32.0)
Race/ethnicity				
Other^b^	61 (3.1)	26 (4.6)	19 (2.4)	16 (2.5)
Hispanic	377 (19.0)	122 (21.5)	137 (17.5)	118 (18.5)
African American	1305 (65.6)	325 (57.2)	524 (66.8)	456 (71.6)
White	246 (12.4)	95 (16.7)	104 (13.3)	47 (7.4)
Age, mean (SD), y	36.9 (8.0)	37.1 (7.8)	37.7 (8.1)	35. 7 (7.9)
Stable housing	1388 (69.8)	427 (75.2)	543 (69.3)	418 (65.6)
Insurance coverage	1695 (85.2)	487 (85.7)	685 (87.4)	523 (82.1)
CD4^+^/100 cells/mm^3^, mean (SD)	4.67 (3.0)	4.77 (3.1)	4.48 (2.9)	4.82 (3.0)
HIV RNA copies/mL, median (IQR)	6200.0 (384.5-41 678.0)	2000 (80-29 000)	8250 (722-51 000)	7700 (920-35 000)
Years in school, mean (SD)	10.30 (4.1)	10.34 (4.3)	10.46 (3.7)	10.05 (4.3)
Depression symptoms: yes (CES-D score ≥16)^c^	998 (50.2)	244 (43.0)	412 (52.6)	342 (53.7)
Drug use^d^	732 (36.8)	168 (29.6)	301 (38.4)	263 (41.3)
Alcohol use, drinks per week				
0-7	1711 (86.0)	503 (88.6)	672 (85.7)	536 (84.1)
>7	242 (12.2)	51 (9.0)	103 (13.1)	88 (13.8)
HIV therapy				
None	815 (41.0)	203 (35.7)	287 (36.61)	325 (51.0)
ART^e^	749 (37.7)	224 (39. 4)	334 (42.6)	191(30.0)
cART	403 (20.3)	139 (24.5)	155 (19.8)	109 (17.1)
Enrollment wave				
1	1255 (63.1)	368 (64.8)	532 (67.9)	355 (55.7)
2	514 (25.8)	169 (29.8)	136 (17.4)	209 (32.8)
3	219 (11.0)	31 (5.5)	115 (14.7)	73 (11.5)
Mortality rate	663 (33.3)	125 (22.0)	288 (36.7)	250 (39.3)
Follow-up, mean (SD), y	13.3 (6.6)	15.1 (6.2)	12.2 (7.1)	13.1 (5.9)
Follow-up, person-years	26 462.7	8570.9	9549.6	8342.2

Abbreviations: ART, antiretroviral therapy; cART, combination antiretroviral therapy; CES-D, Center for Epidemiologic Studies–Depression; IQR, interquartile range.

^a^ Viremia defined as HIV viral load level greater than 200 copies/mL.

^b^ Asian, Pacific Islander, Native American.

^c^ Score of less than 16 indicates no depression; 16 or greater, depression.

^d^ Recreational or illicit, including marijuana or hash; crack; cocaine; heroin; illicit methadone; methamphetamines; amphetamines; narcotics; hallucinogens; and other drugs.

^e^ Medications not constituting cART.

We found statistically significant differences in many characteristics of the women in the various trajectory groups (Table 1). Compared with women in the other 2 groups, those in the high-viremia group were more likely to be African American (456 [71.6%]) or Hispanic (118 [18.5%]), report depressive symptoms (CES-D score ≥16) (342 [53.7%]), have higher rates of current drug (263 [41.3%]) and alcohol (88 [13.8%]) use, be less likely to have stable housing (418 [65.6%]), and be more likely to die (250 [39.2%]). Other characteristics were younger age (mean [SD], 35.7 [7.9] years), lower CD4+ T-lymphocyte counts (mean [SD], 4.82 [3.0] per 100 cells/mm^3^), and unstable housing (418 [65.6%]). There was no significant difference in the groups by mode of HIV transmission risk or by WIHS site.

**Figure 2.**
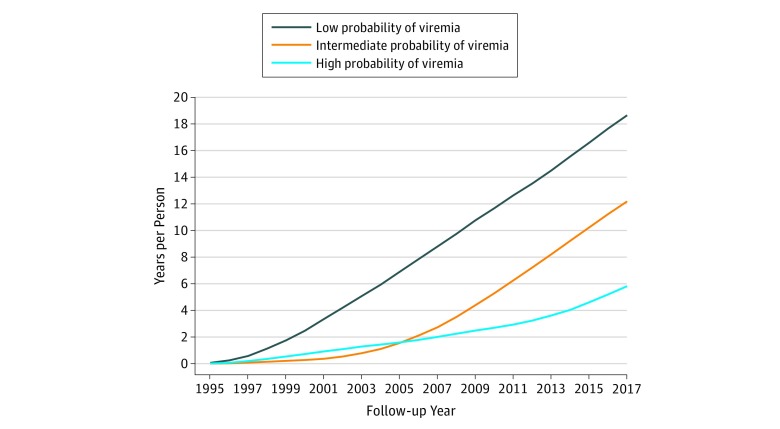
Mean Cumulative Years of HIV RNA Suppression at Less Than 200 Copies/mL Cumulative years of viral suppression were calculated for participants in each trajectory group and the mean cumulative years of viral load suppression was plotted (mean years per participant per group). The longest cumulative period of viral suppression was 18.7 years in the low probability of viremia group, compared with 12.2 years for the intermediate probability of viremia group, and 5.8 years for the high probability of sustained viremia group.

The viral trajectory pattern was dynamic (Figure 1), indicating decreasing probability of having detectable virus for each of the trajectory groups over time; the probability of viremia converged for the intermediate- and low-viremia groups in 2009. Although the high-viremia group showed a decline in viremia over time, the slope of the decline was less pronounced than for the other groups, and this group continued to have high viremia, 41.9% in 2017. These findings are reflected in the mean (SD) cumulative years of viral suppression from 1994 to 2017, which differed for each trajectory group: 18.7 (4.0) years for the low-viremia, 12.2 (3.1) years for the intermediate-viremia, and 5.8 (2.9) years for the high-viremia group (Figure 2).

Mortality varied across the groups, with deaths reported among 125 women (22.0%) in the low-viremia, 288 women (36.7%) in the intermediate-viremia, and 250 women (39.2%) in the high-viremia groups. The overall mortality rate was 31 women per year: 6 in the low-viremia, 13 in the intermediate-viremia, and 12 in the high-viremia groups. Loss to follow-up was 15.9% overall: 21.1% in the low-viremia, 13.4% in the intermediate-viremia, and 14.4% in the high-viremia groups.

**Figure 3.**
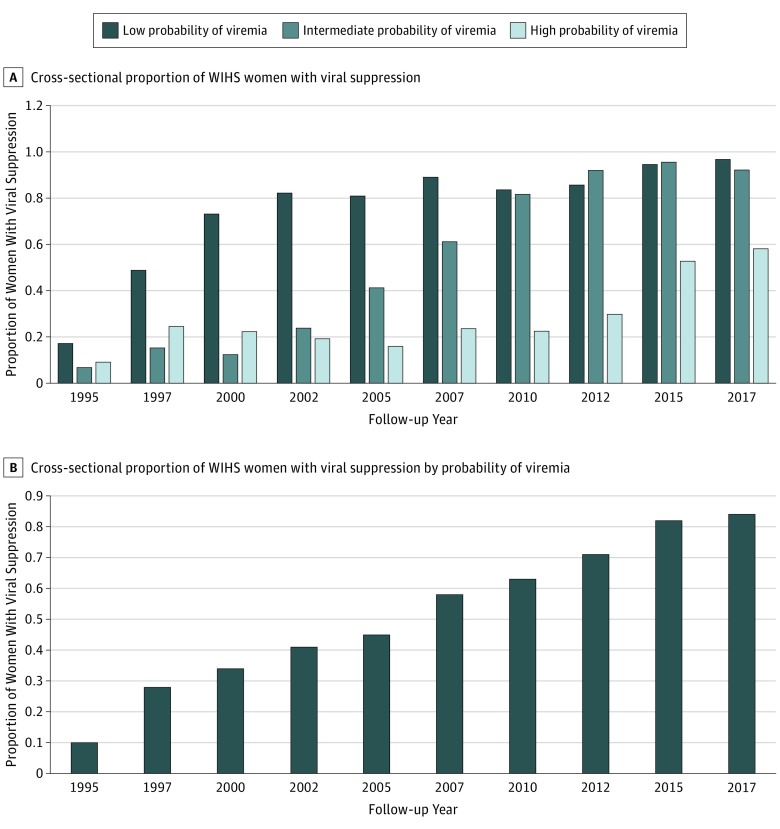
Cross-sectional Viral Suppression Among HIV-Positive Women in the Women’s Interagency HIV Study (WIHS) The proportion of women with viral suppression at 200 copies/mL or less increased over time. A, Cross-sectional proportion of all WIHS women with viral suppression of 200 copies/mL or less. B, Cross-sectional proportion of all WIHS women with viral suppression of 200 copies/mL or less by probability of viremia.

Figure 3 shows the proportion of women with viral suppression using the serial cross-sectional approach often applied in the HIV Care Continuum. The proportion of women with viral suppression improved over time, with 83.4% (346 of 415) of the study population having viral suppression in 2017. However, 71.2% of the women had consistent viral suppression at all visits from 2015 to 2017; 89.6% (310 of 346) had consistent suppression in the low-viremia group and 83.4% (346 of 415) in the intermediate-viremia group, but just 35.2% had consistent suppression in the high-viremia group. The overall median HIV viral load for individuals who were not always virally suppressed from 2015 to 2017 was 16 486 copies/mL: 7286 copies/mL in the low-viremia, 5558 copies/mL in the intermediate-viremia, and 21 817 copies/mL in the high-viremia group.

In the multivariate analysis (Table 2), after adjusting for enrollment wave and HIV treatment, these characteristics were significantly associated with classification in the high-viremia group: African American race (odds ratio [OR], 2.43; 95% CI, 1.75-3.37), Hispanic ethnicity (OR, 1.50; 95% CI, 1.03-2.19), unstable housing (OR, 1.25; 95% CI, 1.03-1.50), lower CD4^+^ T-lymphocyte count (OR, 0.82; 95% CI, 0.80-0.85), depressive symptoms based on CES-D score of 16 or higher (OR, 1.17; 95% CI, 1.01-1.36), drug use (OR, 1.23; 95% CI, 1.01-1.51), not using prescribed medications (OR, 2.59; 95% CI, 1.83-3.65), and less than 95% adherence to antiretroviral therapy (OR, 1.56; 95% CI, 1.37-1.78). Enrollment in the first 2 waves of the study was associated with approximately 50% lower risk of being classified in the high-viremia group. The earliest enrollment was in 2010 for wave 3 enrollees, and cART use varied by wave from 2010 onward: wave 1 (90.5%), wave 2 (86.3%), and wave 3 (81.2%). There was no statistically significant association with enrollment site and viremia.

**Table 2.** Multinomial Regression Analysis for Outcome Variable High Probability of Viremia Trajectory Group, 1994-2017

Outcome Variable	Univariate		Multivariate	
	OR (95% CI)	*P* Value	OR (95% CI)	*P* Value
Race/ethnicity				
White	1 [Reference]		1 [Reference]	
Other^a^	0.85 (0.47-1.56)	.60	0.96 (0.50-1.87)	.93
Hispanic	1.56 (1.23-2.17)	.007	1.50 (1.03-2.19)	.04
African American	2.34 (1.78-3.08)	<.001	2.43 (1.75-3.37)	<.001
Age^b^^,^^c^	0.97 (0.96-0.98)	<.001	0.99 (0.98-0.99)	.03
Stable housing, No. (%)^c^				
Yes	1 [Reference]		1 [Reference]	
No	1.61 (1.39-1.87)	<.001	1.25 (1.03-1.50)	.02
Insurance coverage, No. (%)^c^				
Yes	1 [Reference]	.05	NA	NA
No	1.21 (0.99-1.47)			
CD4^+^/100 cells/mm^3^^c^^,^^d^	0.85 (0.83-0.88)	<.001	0.82 (0.80-0.85)	<.001
Years in school	0.98 (0.95-1.01)	.27	NA	NA
Depression symptoms^c^^,^^e^				
No (CES-D<16)	1 [Reference]	<.001	1 [Reference]	.03
Yes (CES-D≥16)	1.38 (1.23-1.56)		1.17 (1.01-1.36)	
Drug use^c^^,^^f^				
No	1 [Reference]	<.001	1 [Reference]	.04
Yes	1.55 (1.33-1.82)		1.23 (1.01-1.51)	
Alcohol use, drinks/wk^c^				
0-7	1 [Reference]	.001	1 [Reference]	.06
>7	1.61 (1.21-2.14)		1.35 (0.98-1.86)	
HIV therapy^c^				
cART	1 [Reference]		1 [Reference]	
No therapy	2.46 (2.13-2.84)	<.001	1.98 (0.99-3.99)	.05
ART^g^	1.24 (1.09-1.40)	.001	1.08 (0.82-1.43)	.56
Adherence^c^				
≥95%	1 [Reference]		1 [Reference]	
Not taken	3.90 (2.81-5.40)	<.001	2.59 (1.83-3.65)	<.001
<95%	2.16 (1.89-2.46)	<.001	1.56 (1.37-1.78)	<.001
Enrollment wave				
3	1 [Reference]		1 [Reference]	
1	0.64 (0.50-0.81)	<.001	0.47 (0.36-0.63)	<.001
2	0.78 (0.59-1.03)	.08	0.52 (0.38-0.71)	<.001

Abbreviations: ART, antiretroviral therapy; cART, combination antiretroviral therapy; CES-D, Center for Epidemiologic Studies–Depression; NA, not applicable (not included in multivariate analysis); OR, odds ratio.

^a^ Asian, Pacific Islander, Native American.

^b^ With continuous variables, the relative risk is multiplicative per increasing unit change. For example, the OR of viremia greater than 200 copies/mL for age is reduced by a factor of 0.99 for each year of increased age. Therefore, the OR of increased viremia for a women 5 years older than another women would be 0.95 (0.99^5 years^ = 0.95) or 5% less likely to be in the high-viremia group.

^c^ Time-dependent covariates that change in value over visits.

^d^ The OR of increased viremia greater than 200 copies/mL for CD4^+^ T-lymphocyte count level is 0.8 per 100 cells/mm^3^-unit decline. For example the OR of increased viremia for a women with a CD4^+^ T-cell count of 500 cells/mm^3^ compared with one whose level was 200 cells/mm^3^ would be 0.55 (0.82^3^ CD4+/100 = 0.55) or 45% less likely to be in the high-viremia group.

^e^ Score of less than 16 indicates no depression; 16 or greater, depression.

^f^ Recreational or illicit, including marijuana or hash; crack; cocaine; heroin; illicit methadone; methamphetamines; amphetamines; narcotics; hallucinogens; and other drugs.

^g^ Medications not constituting cART.

## Discussion

This study quantified the longitudinal viral trajectories of a cohort of HIV-positive women in the United States and provides a historical perspective on treatment outcomes. When evaluated over the decades of enrollment, just 28.6% of participants were found to have low probability of viremia. However, in the most recent years following a change in treatment guidelines that recommended universal treatment irrespective of CD4^+^ T-lymphocyte count, data from our last study visit window (October 1, 2016, to March 31, 2017) suggest that 84% of women in this study achieved viral suppression. Cross-sectional estimates of HIV viral suppression can miss a participant’s varied experience with viral suppression over time. In reframing the HIV viral outcomes longitudinally over the most recent 2-year time span for which our data are available and allowing for sufficient time for implementation and uptake of the most recent treatment guideline changes in 2012, our data show that 71.2% of women demonstrated sustained viral suppression between 2015 and 2017. Our data agree with recent findings from the US Centers for Disease Control and Prevention that showed a difference in the population with viral suppression that declined from 57.3% using a cross-sectional approach to 47.6% when a longitudinal approach was applied.^10^ Data from the WIHS show that although viral suppression levels have improved over time, a subset of women exists whose levels of viral suppression are not optimized. We were able to further characterize this subset of women using the extensive sociodemographic, clinical, and behavioral data that are routinely collected within the WIHS cohort. Our findings appear to strengthen the call for using longitudinal analyses in assessing viral suppression outcomes to determine the effectiveness of our health care delivery systems and identify key populations with suboptimal treatment outcomes.

Our identification of significant subsets of women with sustained viremia needs to be interpreted in context and partially reflects the evolving guidelines. Treatment initiation used increasingly higher CD4^+^ T-lymphocyte cutoffs as an indication for treatment, for example, the increase of the CD4^+^ T-lymphocyte cutoff from 350 cells/mm^3^ to 500 cells/mm^3^ as an indication for treatment in 2009,^20^ followed by a recommendation for treatment for all individuals irrespective of CD4^+^ T-lymphocyte count in 2012.^21^ This change may partially explain the variations we noted by wave, as participants from earlier periods were more likely to qualify for therapy based on eligibility criteria for treatment until full implementation of the universal treatment guidelines. Although we identified some decline in the probability of viremia among the high-viremia trajectory group, especially since 2012, the persistence of viremia among most participants in this high-viremia group in the most recent years despite guidelines recommending universal treatment raises concern. Demographic, social, and behavioral factors were positively associated with a high probability of viremia, including those that are amenable to interventions, such as depression and drug use. Drug treatment programs have been shown to be associated with improved adherence to antiretroviral therapy.^22^ Treatment for depression has also been associated with improved uptake of antiretroviral therapy and higher odds of achieving viral suppression.^23,24^ However, prior research from our cohort suggests that less than half of the women with clinically relevant symptoms of depression receive treatment, with treatment being even less likely among African American and Hispanic women.^25^ Most publicly funded HIV programs include multidisciplinary teams that aim to address these comorbid conditions. These findings and the association between these factors and long-term viremia underscore the importance of continued program funding to support complementary activities to cART, such as those provided by the Ryan White HIV/AIDS Program.^26^ Further studies are needed to understand how to engage, retain, and effectively treat individuals across racial/ethnic groups to achieve individual as well as national viral suppression goals.

We found an association between unstable housing and high viremia. Several studies have similarly identified stable housing as an important component in achieving viral suppression.^27,28^ Housing voucher and support systems are incorporated into HIV support services and are an important adjunct to treatment services. We found a marginal association of viremia with insurance coverage in the setting of high insurance coverage among our participants. This finding may have been further accentuated had we included participants from our southern sites where insurance coverage is less optimal. We will investigate this association further once sufficient temporal data have been collected from these participants.

This longitudinal approach contributes to our understanding of risks for transmission. There is enthusiasm from policy makers and people with HIV pinpointing undetectable viral load as an effective measure to reduce risk of transmission, described with the colloquialism U = U for undetectable = untransmissible.^29,30^ Even in the most recent years with improved treatment options, our finding that over a quarter of the HIV-positive women do not have consistent viral suppression has potential implications for transmission risk. The median viral load for those without consistent suppression in 2015-2017 across all of the trajectory groups was in the range that could result in a transmission event, including potential transmission of drug-resistant virus. The significant number of women with intermittent viremia across all of the trajectory groups further presents a potential public health challenge. Identification of this phenomenon mirrors previous findings among the Washington, DC, WIHS participants.^12^ This phenomenon supports the need for regionally representative viral load and drug resistance surveillance. Conversely, analysis of our most recent data demonstrates that women without previous viral suppression can achieve and maintain viral suppression. We are conducting further studies using mixed-methods approaches to understand this phenomenon.

This study made use of data collected in a well-characterized cohort with excellent long-term follow-up. These data allowed us to identify important social, biological, clinical, behavioral, and geographic elements associated with lack of viral suppression. As the only national HIV-positive women’s cohort, the WIHS allows us to study long-term outcomes in women who compose 24% of the HIV population in the United States.^31^ As such, nationwide data may not identify or highlight important elements that uniquely affect women.

### Limitations

Limitations of the group-based trajectory analysis approach include groupings that may homogenize potential subgroups. However, the high posterior probabilities suggest high precision of group assignments.^19^ The individual-based quantitation of cumulative years of viral suppression and incremental increase in cumulative viral suppression years that we identified across trajectory groups substantiate our approach. We also identified enrollment wave as being significantly associated with the high viremia level. This finding is partially explained by differences in therapy and may also be affected by shorter follow-up and fewer data points available for wave 3 enrollees. The viremia trajectories and associated findings may also be affected by survival bias with those who remain active in the cohort from the earlier waves being those who accessed and adhered to treatment. However, these participants likely are representative of HIV-positive individuals in the community who acquired HIV during the first 2 decades of the epidemic.

## Conclusions

Our findings suggest the utility of a probability-based longitudinal approach to characterize viremia patterns and identify factors associated with different long-term viremia patterns. This longitudinal approach appears to provide a more accurate portrayal of viremia outcomes than the cross-sectional HIV care continuum.^1^ Given geographic differences in health care access and regional burden of HIV, we are planning further analyses that will focus on recently recruited southern WIHS participants after they have been followed up for sufficient time to allow for an accurate longitudinal depiction of their viremia trajectories. We also recommend exploring health care models to identify programmatic elements that can be replicated to improve longitudinal viral suppression.

## Notes

Supplement. **eTable.** Criteria for Trajectory Group Selection **eFigure.** Viral Trajectories for HIV-Positive Women in the WIHS-HIV RNA>1000 copies/mL
