# Supplementary material for: Viremia Trajectories of HIV in HIV-Positive Women in the United States, 1994-2017
Source: JAMA Netw Open. 2019 May 17;2(5):e193822. doi: 10.1001/jamanetworkopen.2019.3822 (PMC6537820; doi:10.1001/jamanetworkopen.2019.3822)
Supplement: Supplement. — eTable. Criteria for Trajectory Group Selection eFigure. Viral Trajectories for HIV-Positive Women in the WIHS-HIV RNA>1000 copies/mL [file jamanetwopen-2-e193822-s001.pdf]

Supplementary Online Content

Kassaye SG, Wang C, Ocampo JMF, et al. Viremia trajectories of HIV in HIV-positive women in the United States, 1994-2017. *JAMA Netw Open*. 2019;2(5): e193822. doi:10.1001/jamanetworkopen.2019.3822

eTable. Criteria for Trajectory Group Selection

eFigure. Viral Trajectories for HIV-Positive Women in the WIHS-HIV RNA>1000 copies/mL

This supplementary material has been provided by the authors to give readers additional information about their work.

© 2019 Kassaye SG et al. *JAMA Network Open.*

eTable. Criteria for trajectory group selection

Selection of optimal number of trajectory groups was guided by Nagin's criteria:

(1) Substantive theory, (2) Bayesian Information Criteria (BIC) , (3) Average Posterior Probabilities, and (4) group size

	Number of groups	Polynomial order	BIC	Average Posterior Probability
Model 1	2	3, 3	-23168	0.96, 0.95
**Model 2**	**3**	**3,3,3**	**-22060**	**0.92, 0.75, 0.88**
Model 3	4	3,3,3,3	-21416	0.87, 0.82, 0.84, 0.76

Model 1 would have the best fit if we only apply the statistical method to assess model fit. Model 2 has a lower BIC than Model 3, with higher average posterior probability support for each group than model 3. It also captures the heterogeneity of viremia outcomes that we have observed from 1994-2017. Thus, we selected model 2, the three-group trajectory as the best fit for our data and the analyses undertaken in this manuscript.

© 2019 Kassaye SG et al. *JAMA Network Open.*

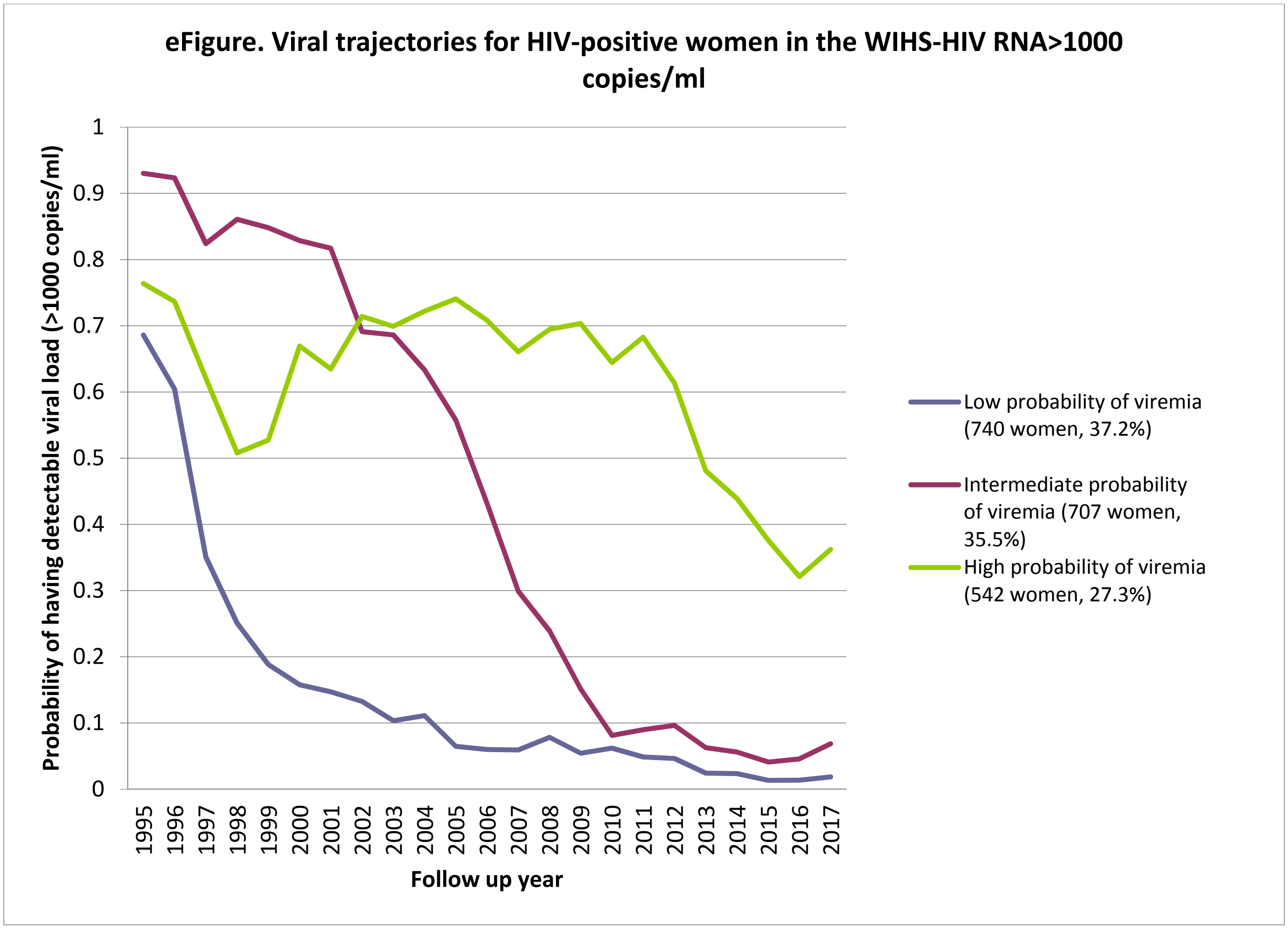

eFigure 1. Group-based trajectory analysis identified three distinct viral trajectories among participants in the WIHS when using a viremia cutoff >1000 copies/mL. The low probability of viremia group consisted of 740 women, representing 37.2% of the total population; 707 women were in the intermediate probability of viremia group, representing 35.5% of the total population; while 542 women, 27.3% of the population, had high probability of viremia over the 23-year time period.

© 2019 Kassaye SG et al. *JAMA Network Open.*
